# Unveiling the Probiotic Potential of *Streptococcus thermophilus* MCC0200: Insights from In Vitro Studies Corroborated with Genome Analysis

**DOI:** 10.3390/microorganisms12020347

**Published:** 2024-02-07

**Authors:** Neelam Kapse, Vaidehi Pisu, Tanisha Dhakephalkar, Prajakta Margale, Deepa Shetty, Shilpa Wagh, Sumit Dagar, Prashant K. Dhakephalkar

**Affiliations:** 1 Bioenergy Group, MACS-Agharkar Research Institute, Gopal Ganesh Agarkar Road, Pune 411004, Maharashtra, India; neelamkapse@aripune.org (N.K.); vaidehipisu@aripune.org (V.P.); deepashetty@aripune.org (D.S.);; 2 Department of Microbiology, Savitribai Phule Pune University, Ganeshkhind Rd., Aundh, Pune 411007, Maharashtra, India; 3 Hi Tech BioSciences India Ltd., Research & Development Centre, Plot No. 6 & 8, Ambadvet Industrial Estate, PO Paud, Pune 412108, Maharashtra, India

**Keywords:** *Streptococcus thermophilus*, gastrointestinal transit, adhesion, health-promoting, anti-hypercholesterolemic activity, GRAS, genome

## Abstract

*Streptococcus thermophilus* is widely used as a starter culture in the dairy industry and has garnered attention as a beneficial bacterium owing to its health-promoting functionalities in humans. In this study, the probiotic potential of *S. thermophilus* MCC0200 isolated from a dairy product was investigated through a combinatorial approach of in vitro and in silico studies. MCC0200 demonstrated the ability to survive harsh gastrointestinal (GI) transit, adhere to intestinal mucosa and exert health-promoting traits in in vitro studies. These findings were corroborated with in silico evidence, wherein, MCC0200 genome harboured genes associated with tolerance to GI conditions, intestinal adhesion and colonization. Genome mapping also highlighted the ability of MCC0200 to produce compounds advantageous for the host (folate, bacteriocins), to release antioxidant enzymes that can quench the free radicals (superoxide dismutase, NADH peroxidase), and to metabolize food components that can be harmful to sensitive people (lactose). MCC0200 also demonstrated a positive effect on reducing cholesterol levels, proving to be a potential candidate for food and pharmaceutical applications. The absence of transmissible antibiotic resistance genes and virulence genes underscored the generally regarded as safe (GRAS) nature of MCC0200. This study explored the potential of *Streptococcus thermophilus* for its probable applications as a probiotic beyond the dairy industry.

## 1. Introduction

Probiotics, live microorganisms with potential health benefits, have gained significant attention in the field of microbiology and human health. Among the diverse range of probiotic strains, *Streptococcus thermophilus* has emerged as a thermophilic species of great interest. *S. thermophilus* has been extensively utilized as a starter culture in the dairy sector as well as in many traditional fermented products, including yogurt, along with *Lactobacillus delbrueckii* subsp. *bulgaricus* [1]. It is the second most important species among industrial lactic acid bacteria after *Lactococcus lactis* [2].

*Streptococcus thermophilus* is a Gram-positive bacterium classified under the phylum *Firmicutes* and the family *Streptococcaceae*. *S. thermophilus* is the only species within the *Streptococcus* genus (which primarily consists of commensals and pathogenic species), that has been given the generally recognised as safe (GRAS) status by the Food and Drug Administration [FDA] [3], and the qualified presumption of safety (QPS) status by the European Food Safety Authority [EFSA] [4]. Numerous investigations have sought to elucidate the genetic underpinnings that govern the physiological and metabolic characteristics of *S. thermophilus*, with a primary focus on delineating its technological capabilities. Commonly studied technological aspects of *S. thermophilus* include milk acidification, lactose and galactose utilization, proteolytic activity, and exopolysaccharide (EPS) production [5].

A variety of probiotic products can be found in the market featuring *S. thermophilus* biomasses under different brand names, including Fermental, Floratrex, Neuflor, Multibiotics, Perfect Biotics, Probioguard, Visbiome, VSL#3, Yovis, etc. [6]. However, there are still uncertainties about designating this species itself as a probiotic, as the data regarding its ability to survive gastric transit and exert beneficial effects in the human gut are not unequivocal [7]. Several studies have reported no viable *S. thermophilus* cells in the faecal samples of healthy adults fed with pasteurized or fresh yogurt, indicating their sensitivity to gastro-intestinal transit in humans [8,9]. Owing to the sensitivity of *S. thermophilus* to gastrointestinal conditions, its probiotic status remains a topic of ongoing debate and investigation. On the contrary, a few studies have established that *S. thermophilus* had the capacity to survive the passage through the gastro-intestinal tract [10,11]. Despite these challenges, it is important to note that *S. thermophilus* still possesses certain probiotic properties and has been associated with potential health benefits. Its ability to produce antimicrobial substances, compete with pathogenic bacteria, and modulate the immune system suggests that it may positively impact gut health [12]. However, the extent of these effects and the strain-specific variations in probiotic potential require further investigation. Comprehensive studies integrating in vitro assessments and genomic analysis are essential to address the concerns surrounding the probiotic status of *S. thermophilus*. These investigations can provide a deeper understanding of the strain’s survival mechanisms, interaction with the gut environment, and potential health benefits.

Over the last two decades, numerous genomes of *S. thermophilus* have been published, significantly enhancing our comprehension of the molecular-level metabolic activities of this bacterium [13]. These activities encompass EPS and folate biosynthesis [2,14,15], resistance to bacteriophages [16], proteolytic systems [17], and carbohydrate metabolism [18], among others. Most of these functionalities are strain-specific, indicating that the diverse spectrum of health-promoting attributes exhibited by *S. thermophilus* contributes to a considerable variation in the genomic content among strains. Genomic-level analysis is essential for a more comprehensive understanding of the distinctive features of each strain. Moreover, the comparative genomic analysis of diverse *S. thermophilus* strains exhibiting various technological properties has contributed to an enhanced understanding of the correlation between genetic characteristics and phenotypic traits [19,20]. However, each study has illustrated only a limited number of probiotic traits of *S. thermophilus*. Strain-to-strain variation was also not addressed in most of these studies.

In this manuscript, we present a comprehensive investigation to explore the probiotic properties of *S. thermophilus*. The study encompasses a combination of rigorous in vitro assessments and detailed genomic analysis to shed light on this thermophilic species’ multifaceted capabilities and health-promoting attributes. This study established *S. thermophilus* MCC0200 as a safe probiotic candidate with diverse health-promoting traits, providing essential information for its potential utilization as a probiotic in contexts beyond the dairy industry.

## 2. Materials and Methods

### 2.1. Bacterial Strain and Culture Conditions

The bacterial culture *Streptococcus thermophilus* MCC0200 was isolated from a dairy product, i.e., hung curd locally known as ‘Chakka’ and cultured in brain heart infusion (BHI) medium (HiMedia: M210,Mumbai, India) supplemented with 1% sucrose (SRL: 84973, Mumbai, India) at 37 °C. Stock culture was preserved in BHI broth mixed with 20% glycerol at −80 °C. MCC0200 has been deposited in the National Centre for Microbial Resource (NCMR) in Pune, India.

### 2.2. Genome Sequencing and Annotation

Whole genome sequencing of MCC0200 was performed on both an Illumina HiSeq platform and an Oxford nanopore (flow cell FLO-MIN106D) platform. The genome assembly of MCC0200 was performed using Unicycler 0.5.0. The genome quality of MCC0200 was evaluated using CheckM v1.0.7 tool [21]. The general functional annotation of MCC0200 was carried out using rapid annotations using subsystems technology (RAST) server [22] and KEGG (Kyoto Encyclopedia of Genes and Genomes) tool [23].

### 2.3. Evolutionary Analysis

The phylogeny of MCC0200 was analysed using AutoMLST, an automated web tool. In silico DNA–DNA hybridization (DDH) and Average Nucleotide Identity (ANI) between closely-related species was calculated using the genome to genome distance calculator (GGDC) (http://ggdc.dsmz.de/home.php (accessed on 25 July 2022) and Average Nucleotide Identity calculator (ANI), EZBiocloud. Further, BLAST ring image generator (BRIG) analysis was performed for circular genome comparison of MCC0200 with other *S. thermophilus* strains.

### 2.4. Nucleotide Sequence Accession Number

This whole genome shotgun project has been deposited at DDBJ/ENA/GenBank under the accession JAVCAM000000000. The version described in this paper is version JAVCAM010000000.

### 2.5. In Vitro Evaluation of Probiotic Properties of MCC0200

#### 2.5.1. Resistance to Simulated Gastrointestinal Conditions

The resilience of MCC0200 to harsh gastro-intestinal conditions was examined as previously described by Vecchione et al., 2018 [24], with certain modifications. A total of 100 µL of MCC0200 cells were re-suspended in 5 mL of simulated gastric fluid (SGF) (Composition of SGF: 0.03 M Sodium chloride (SRL: 41721, Mumbai, India), 0.084 M Hydrochloric acid (SDFCL: 20125 L25, Mumbai, India), and 0.32% (*w*/*v*) of pepsin (HiMedia: GRM084, Mumbai, India)) of pH 2.5 with the initial bacterial count of ~10^9^ cel1s/mL and incubated at 37 °C for 0, 30, 60, and 120 min. The viable cell count was determined using the standard plate count method by plating 100 μL of each serially diluted cell suspension on brain heart infusion (BHI) agar plates. Simulated intestinal fluid (SIF) comprising of 0.3% *w*/*v* ox gall bile salts (HiMedia: RM010, Mumbai, India) and 0.1% *w*/*v* pancreatin (HiMedia: RM7384, Mumbai, India) prepared in sterile 0.85% saline solution of pH 8.0 was used to assess the bile tolerance of MCC0200. An aliquot of 100 µL of 10^9^ cells/mL of MCC0200 were inoculated in 5 mL of simulated intestinal fluid and incubated at 37 °C for 0, 30, 60, 120, 240, and 360 min. At each time point, aliquots (100 µL) of the microbial suspension were serially diluted and seeded on BHI agar.

#### 2.5.2. Adhesion Potential

##### Cell Surface Hydrophobicity

The cell surface hydrophobicity of MCC0200 was determined using the bacterial adherence to hydrocarbons (BATH) assay [25,26]. Briefly, 3 mL of MCC0200 cell suspension (ODi ~1) was mixed with 1 mL of hydrocarbon (Chloroform (SRL: 0322123, Mumbai, India), ethyl acetate (Fisher Scientific: 43536, Waltham, MA, USA) and xylene (SRL: 242921, Mumbai, India)), incubated at 37 °C for 10 min, vortexed for 15s and allowed to stand undisturbed at 37 °C for 30 min for phase separation. The lower aqueous phase was collected carefully, and OD600 was recorded as ODt. Percent hydrophobicity (adherence of cells to hydrocarbons) was calculated by using the following formula: % Hydrophobicity = (ODi – ODt/ODi) × 100.

##### Aggregation Assay

For the auto-aggregation assay, 1 mL of MCC0200 cell suspension with OD600 of ~1.0 (A0) was dispensed in tubes, vortexed and incubated at 37 °C under static conditions. Absorbance (600 nm) was recorded at 1 h, 2 h, 3 h and 4 h intervals by carefully withdrawing the supernatant (At). For the co-aggregation assay, the MCC0200 cell suspension and the cell suspension of pathogens, namely, *Staphylococcus* aureus ATCC 6538, *Enterococcus faecalis* ATCC 29212, *Escherichia coli* ATCC 8739, *Enterobacter aerogenes* ATCC 13048, *Salmonella typhimurium* ATCC 13311, *Shigella dysenteriae* ATCC 13313, *Klebsiella pneumoniae* ATCC 13883 and *Pseudomonas aeruginosa* ATCC 10145 with an optical density of 1.0 at 600 nm was prepared. Equal volumes of MCC0200 (ODCul) and pathogen cell suspension (ODPath) were mixed and vortexed. Axenic bacterial cultures were used as controls. The tubes were incubated at 37 °C under static conditions and the absorbance (600 nm) was monitored at 1 h and 4 h intervals. Percentage auto-aggregation and co-aggregation were determined using the formula:

% Auto-aggregation = (1 − At /A0) × 100

% Co-aggregation = [(ODPath+ ODCul) − ODMix/(ODPath + ODCul)] × 100

##### In Vitro Binding to Mucin, Fibrinogen and Collagen

MCC0200 was assayed for binding to different substrates immobilized on 96-well plates. Plates were covered with the different substrates (200 µL) overnight at 4 °C. Mucin (500 µg/mL) (HiMedia: RM8678, Mumbai, India), fibrinogen (50 µg/mL) (HiMedia: RM4279, Mumbai, India) in 50 mM carbonate/bicarbonate buffer pH 9.6, and collagen (50 µg/mL) (HiMedia: TC343, Mumbai, India) in PBS pH 5.5 were used. After immobilization, wells were washed three times with PBS and blocked for 2 h with BSA. A total of 200 µL of MCC0200 was added to each well in PBS adjusted to an OD550 nm of 1 and plates were incubated overnight at 4 °C. Non-adhered cells were removed by washing three times with 200 µL of PBS plus 0.05% Tween 20 (MP Biomedicals: 103168, Santa Ana, CA, USA) and the plates were dried at 55 °C. Adhered cells were stained with crystal violet 1 mg/mL (200 µL/well) for 45 min. After six washes with PBS, the colorant was liberated with 50 mM citrate buffer pH 4.0 (200 µL /well) for 45 min and the absorbance at 595 nm. The adherence to mucus is an inducible trait known to be triggered in the presence of mucin in many probiotic strains. Thus, to assess the effect of mucin on the adhesion ability of MCC0200, bacterial cells were also grown in MRS broth supplemented with 0.1% mucin. BSA coated wells were used as control.

##### Adhesion of MCC0200 to HT-29

The ability of MCC0200 to adhere to the human intestinal cell line: HT-29 was investigated, as previously described by Sharma and Kanwar, 2017 [27]. HT-29, a human colorectal adenocarcinoma cell line was purchased from NCCS (Pune, India) with ATCC no. HTB–38 and maintained in Dulbecco’s modified eagle medium (DMEM, Gibco: 11965092, Waltham, MA, USA). HT-29 dells were seeded at concentration of 1 × 10^5^ cells/mL in 24-well tissue culture plates containing coverslips and incubated until a monolayer was formed. The tissue culture plate with ready monolayer was incubated with antibiotic and FBS free DMEM for 30 min and further used for adhesion assay. For the adhesion assay, 1 mL of MCC0200 (10^8^ cells/mL) was added to tissue culture plate wells containing the HT-29 cells and allowed to incubate at 37 °C for 2 h to mediate adherence. After incubation, each well was gently washed with phosphate buffered saline (PBS) twice, to remove non-adhered bacteria and fixed with 2% glutaraldehyde and processed. During the experiment, wells containing only HT-29 cells were used as controls. Each assay was performed in duplicate to determine inter-assay variation. The observation of the adhesion of MCC0200 on HT-29 cells was completed using scanning electron microscopy (SEM) as described by Inturri et al., 2014 [28]. Twenty random fields were captured for counting the number of adhered bacteria per animal cell. The final results were expressed as no. of bacterial cells per 100 HT-29 cells.

### 2.6. Antioxidant Activity

The intact cells of MCC0200, grown at 37 °C overnight in BHIB, were harvested through centrifugation 8000× *g* for 5 min at 4 °C. The cells were washed with phosphate buffered saline (PBS) and suspended in PBS to adjust the concentration to 10^9^ cells/mL, which was used as the bacterial suspension.

#### 2.6.1. Scavenging Activity to 2,2-Diphenyl-1-Picrylhydrazyl Free Radical (DPPH)

The antioxidative potential of MCC0200 was assessed by measuring its DPPH free radical scavenging activity according to the method of Mu et al., 2018 [29], with some modifications. Briefly, 1.0 mL of the sample (bacterial cells suspended in PBS) was added to 1.0 mL of DPPH (HiMedia: RM2798, Mumbai, India)–ethanol (Emcure: 1.00983.0, Pune, India) solution (0.2 mM). The DPPH–ethanol solution was prepared as follows: 78.86 mg of DPPH powder was added to 100 mL ethanol to prepare main stock of DPPH–ethanol solution. Subsequently, the working stock of 0.2 mM DPPH–ethanol solution was prepared by mixing 10 mL of main stock with 40 mL of ethanol and 50 mL D/W. The mixture was mixed and incubated at 25 °C in the dark for 30 min. The control group included PBS and DPPH–ethanol solution. The blank group contained sample and ethanol. The optical absorbance at 517 nm of supernatant was measured in triplicate. Ascorbic acid (10 µg) was used as the positive control. The DPPH scavenging activity was defined as

Scavenging Activity (%) = [1 − (A_sample_ − A_blank_)]/A_control_] × 100

where, A_sample_ is the optical absorbance at 517 nm of the sample group, A_blank_ is the optical absorbance at 517 nm of the blank group, and A_control_ is the absorbance of the control group.

#### 2.6.2. Scavenging Activity to ABTS (2,2′-Azino-Bis(3-Ethylbenzothiazoline-6-Sulfonic acid) Radical

The ABTS radical scavenging activity was determined as described in Yan et al., 2018 [30]. Briefly, 50 µL of the sample (bacterial cells suspended in PBS) was added to 3 mL of the diluted ABTS solution The ABTS solution was prepared by mixing equal volumes of 7 mM ABTS stock solution (HiMedia: RM9270, Mumbai, India) with a 2.45 mM potassium persulfate solution (HiMedia: GRM7412, Mumbai, India). Subsequently, the mixture was stored in dark at room temperature for 12–16 h. Following this incubation period, the ABTS solution was appropriately diluted with 10 mM phosphate-buffered saline (PBS, pH 7.4) to an absorbance of 0.70 ± 0.02 at 734 nm. The tubes were incubated for 6 min at room temperature in the dark. The absorbance of the mixture was immediately measured at 734 nm. The blank group contained sample with PBS; the control was prepared using distilled water and ABTS reagent. The ABTS radical scavenging activity (%) was calculated as follows:

Scavenging Activity (%) = [1 − (A_sample_ − A_blank_)]/A_control_] × 100

where, A_sample_ is the optical absorbance at 734 nm of the sample group, A_blank_ is the optical absorbance at 734 nm of the blank group, and A_control_ is the absorbance of the control group.

### 2.7. In Vitro Evaluation of the Anti-Hypercholesterolemic Effect of MCC0200

The anti-hypercholesterolemic activity of MCC0200 was tested using the method of Tomaro et al., 2014 [31]. Briefly, 1% of overnight grown culture of MCC0200 (cell density: 1.1 × 10^8^ cells/mL) was inoculated in 10 mL of brain heart infusion broth + 1% sucrose prepared in SIF (simulate intestinal fluid: 0.3% ox bile, 0.1% pancreatin prepared in sterile 0.85% saline solution, pH 8.0) + 100 µg/mL of water soluble cholesterol (SIGMA ALDRICH: C1145-16, St. Louis, MO, USA) and incubated at 37 °C for 24 h. The above mixture was then centrifuged at 5500× *g* for 15 min at 4 °C, and 1 mL of supernatant was collected for further analysis. Residual cholesterol in the spent broth was determined using the O-phthaldehyde method described by Rudel and Morris (1973) [32]. The cholesterol assimilated was determined by the difference between cholesterol level in the 0 h and 24 h time frame.

% Cholesterol Assimilated = [Cholesterol Assimilated (μg/mL)/Cholesterol at 0 h (μg/mL)] × 100.

### 2.8. Screening of MCC0200 for Beta-Galactosidase Production

The production of beta-galactosidase by MCC0200 was determined using substrate hydrolysis method, wherein, 10µL of overnight grown culture of MCC0200 was spot inoculated on cystine–lactose–electrolyte-deficient (CLED) agar (0.4% peptone Type I (HiMedia: RM667, Mumbai, India), 0.4% Tryptone (HiMedia: CR014, Mumbai, India), 0.3% beef extract (HiMedia: CR002), 0.012% L-cystine, 0.002% bromothymol blue, pH 7.0)) plates supplemented with 1% lactose (HiMedia-25957, Mumbai, India). The plates were then incubated at 37 °C for 24 to 48 h. The medium incorporates bromothymol blue as an indicator that transitions to yellow under acidic pH conditions and to a blue shade under alkaline pH conditions. Lactose, being a fermentable sugar, undergoes hydrolysis if the test strain produces enzymes like beta-galactosidase. This enzymatic activity results in the formation of lactic acid, causing a reduction in pH, which is manifested by a yellow colour in the bacterial growth.

### 2.9. Safety Assessment

The safety assessment of MCC0200 was performed as per EFSA guidelines (EFSA FEEDAP Panel, 2018) [33] as elucidated in the following sections.

#### 2.9.1. Antibiotic Susceptibility/Resistance Testing

The antibiotic susceptibility/resistance of MCC0200 was determined by using E-test strips (Ezy MIC strips, HiMedia, Mumbai, India). The antibiotics were chosen in accordance with the EFSA document addressing bacteria of human significance. The E-test strips of ampicillin, vancomycin, clindamycin, chloramphenicol, streptomycin, gentamicin and tetracycline were used in the concentration range 0.016–256 µg/mL. Briefly, 100 µL of bacterial suspension (10^8^ cells/mL) was spread onto BHI agar media. E-test strip of each antibiotic was placed at the centre of each agar plate and incubated for 24 h at 37 °C. The lowest concentration of antibiotic that inhibited the visible growth of MCC0200 was determined as MIC. The strain was categorized as susceptible or resistant to the antibiotic tested based on the microbiological cut-off values published by EFSA. The presence of antibiotic resistance associated genes in MCC0200 genome were predicted using the online tools: resistance gene identifier (RGI) version 5.1.1 of the comprehensive antibiotic resistance database (CARD) version 3.1.0 [34] and the ResFinder 4.1 database [35].

#### 2.9.2. Pathogenicity and Virulence

The virulence factors in MCC0200 genome were detected using Virulencefinder v2.0 and the pathogenicity was predicted using PathogenFinder v1.1 web tool.

#### 2.9.3. Stability of the Genome

Genome stability was investigated to determine the probability of mobilization of transferrable genetic elements (if present) among strains. Prophage sequences were investigated using PHASTER web-based server [36]. For prediction of (CRISPR) and cas genes, CRISPRCasFinder tool [37] was used. Plasmids were screened using PlasmidFinder version 2.1 [38].

## 3. Results and Discussion

The strain specificity of probiotic attributes is well-established, serving as a significant impetus for the continual exploration of more efficacious strains. In pursuit of this objective, the current investigation was initiated to assess the probiotic potential of *S. thermophilus* MCC0200, isolated from hung curd, employing a combinatorial approach encompassing in vitro studies and genomic analysis.

### 3.1. Genome Attributes of S. thermophilus MCC0200:

*De novo* genome assembly of *S. thermophilus* MCC0200 resulted in a circular chromosome of 1,855,815 bp with an average GC content of 39.1% (Table 1). Our data was found to be in congruence with the published study on comparative genome analysis of 23 *S. thermophilus* strains, wherein the chromosome length ranged between 1.73 to 2.10 Mbp with average GC content of 39.0% [39]. In total, 100% genome completeness was achieved, as per CheckM tool. RAST annotation of MCC0200 genome revealed a total of 218 subsystems encoding 2239 coding sequences (CDS) and 83 RNA encoding genes. The protein coding genes reported in other *S. thermophilus* strains was between 1555 and 1854. The differences in genome size and protein coding genes suggested significant variations in both gene gain and gene loss events throughout the evolutionary history of the distinct strains [39].

### 3.2. Evolutionary Analysis and Comparison of MCC0200 with Other S. thermophilus Strains

The multilocus sequence analysis (MLSA) was conducted to elucidate the phylogenetic relationships within *Streptococcus* sp. utilizing an automated webserver. The phylogenetic tree was constructed based on multiple core genes using the autoMLST alignments resulted in clustering of MCC0200 with *S. thermophilus* strains LMD-9, TH1477, MTH17CL396, TH1436 and TH1435 indicating their high degree of evolutionary relatedness (Figure S1).

The phylogeny of MCC0200 was further resolved through in silico DNA–DNA hybridization (DDH) and Average Nucleotide Identity (ANI) analysis. The established threshold values for DDH and ANI to denote the same species are 75% [40] and 95% [41], respectively. The genome of MCC0200 showed a maximum similarity with strain LMD-9 with the DDH (%) and ANI (%) score of 99.70% and 99.93%, respectively, ascertaining the relatedness between them. The DDH and ANI scores obtained after analysis with various strains are given in Table 2.

**Figure 1:**
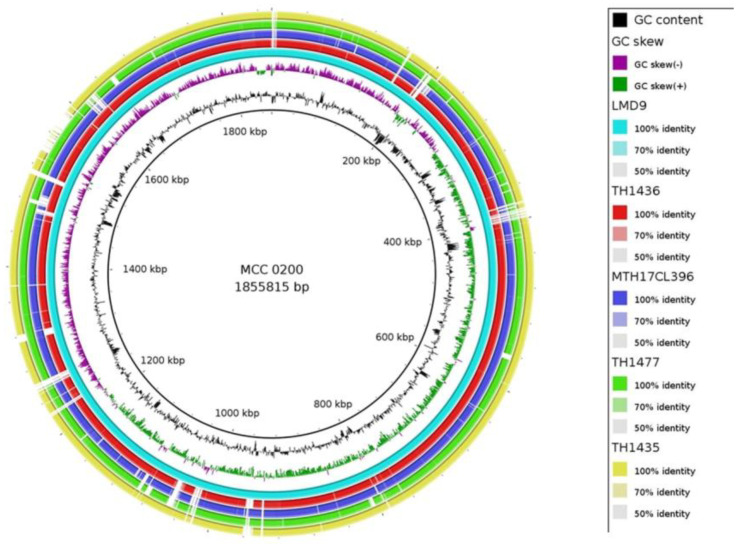
Circular genome comparison of MCC0200 with other *S. thermophilus* strains. Each coloured ring represents a query genome. MCC0200 genome was used as a reference, by running BLASTn in BRIG software (version: 0.95). The intensity of the colour indicates relative levels of nucleotide homology between the reference and query genomes.

BLAST ring image generator (BRIG) analysis [42] generated a circular image comparing the publicly available *S. thermophilus* genomes with MCC0200 (Figure 1). The gaps in the circular image between the reference MCC0200 and the query genomes of LMD-9, TH1477, MTH17CL396, TH1436 and TH1435, indicated the differences between the strains of *S. thermophilus*, indicating MCC0200 to be a different strain of *S. thermophilus*.

### 3.3. Assessment of Probiotic Properties

#### 3.3.1. Resistance to Gastric Conditions

Survival during transit through the gastrointestinal tract is a critical aspect for a probiotic bacterium to effectively confer benefits to the host and hence, must be rigorously assessed. Several investigations have reported contradictory findings regarding the probiotic potential of *S. thermophilus*, owing to its sensitivity to GI tract conditions [6]. The viability of *S. thermophilus* following passage through the digestive system still remains a subject of debate for certain researchers. The present study assessed the viability of MCC0200 after exposing it to simulated gastric juice of pH 2.5. MCC0200 could tolerate simulated gastric juice up to 60 min displaying 5 log reduction in the viable count from 3.75 ± 10 × 10^7^ CFU/ ml to 1.5 ± 0 × 10^2^ CFU/mL (Table S1). Recent studies have presented various degrees of gastric tolerance of *S. thermophilus* strains. Zhang et al., 2020 [43], studied the gastric tolerance of 10 strains of *S. thermophilus* in SGF of different pH ranging from pH 2.0 to pH 7.0, wherein, at pH 2.0, only two strains had a survival rate above 30%. In another study, no viable cells of *S. thermophilus* were detected after 1.0 h of exposure to SGF of pH 2.5 [44].

To gain mechanistic insights into the GI tract survival strategy of MCC0200, its genome was mined for the marker genes associated with gastric stress tolerance, which revealed the presence of an arsenal of genes contributing to the acid tolerance (Table 3). Presence of such diverse genes indicated the mechanism of acid stress resistance in MCC0200 to be multifarious, ensuring its survival during the gastrointestinal transit. Genes encoding for proteins F**0**-F**1** ATPase proton pump and sodium/proton antiporters were detected. F**0**-F**1** ATPase proton pump regulates cytoplasmic pH efficiently, utilizing ATP hydrolysis to pump H^+^ out of cells. This process helps maintain pH homeostasis, protecting cells from damage induced by acidic environments. Studies on *S. thermophilus* LMD-9 have revealed the involvement of proton translocating F0F1-ATPase system in response mechanism to acid stress [45]. Na^+^/H^+^ antiporters contribute to cytoplasmic pH homeostasis by allowing exchange of protons for Na^+^ ions generated across the cell membrane by specific transporters, such as ion-pumping ATPases [46]. Additionally, genes encoding ureI, structural (*ureABC*) and accessory (*ureEFGD*) genes were detected in MCC0200 genome, indicating a probable mechanism for acid tolerance. The urease system produces NH_3_ and CO_2_ from urea, providing protection against acid stress. This system has been extensively studied in *S. thermophilus*, and *S. salivarius* [47].

In addition, genes involved in repair of damaged proteins and DNA to resist acid stress such as *DnaK*, *DnaJ*, *GrpE*, *HrcA*, *GroEL*, *GroES*, Clp proteases, and *EF-Tu*, *recA*, *UvrABCD*, DNA polymerase, and DNA ligase, etc., were also detected.

Presence of key genes in major pH homeostasis pathway of MCC0200 validates the gastric tolerance observed in in vitro studies. Our findings differ from the available literature, wherein, several *S. thermophilus* strains were found to be sensitive to harsh conditions of GI transit, naming them as a transient probiotic [2]. Conversely, our study asserts that gastric tolerance is a strain-specific trait and, therefore, cannot be universally generalized.

Bacteria commonly encounter stress induced by bile acids upon entering the small intestine. The impact of bile acids on bacterial viability has been observed through the compromise of cell membrane integrity, leading to reduced bacterial survival [48]. Thus, in this study, bile tolerance of MCC0200 was assessed in SIF containing 0.3% ox gall. MCC0200 demonstrated remarkable tolerance to SIF for up to 360 min (Table S2), exhibiting a viable count of 1.1 ± 0.28 × 10^7^ CFU/mL with only 1 log reduction in viability, suggesting the strain’s robustness to survive in bile stress prevalent in GI tract. Our findings were in congruence with the reported literature, wherein, different studies have reported efficient bile tolerance (up to 1% and in some cases even up to 2%) trait of *S. thermophilus* [2,49,50].

Bile salt hydrolases and choloyglycine hydrolases are the key enzymes known to confer bile salt resistance in bacteria [51]. Intriguingly, the MCC0200 genome did not contain genes encoding these key proteins associated with bile resistance. This absence indicates the involvement of alternative tolerance mechanisms. Studies on *S. thermophilus* LMD-9 strain, demonstrated the involvement of cell surface proteins in withstanding the detrimental effects of bile salts by maintaining the cell membrane architecture and integrity. Notably, sortase A (*SrtA*) and sortase-dependent proteins (SDPs), such as cyclic-nucleotide phosphodiesterase, have been implicated in resisting bile salts, alongside their recognized role in adhering to intestinal epithelial cells (IECs). The absence of *SDP* at the cell surface could increase cell membrane permeabilization of LMD-9 strain to bile salts, rendering the LMD-9 strain more susceptible to bile salts. These *SDPs* contribute to resistance to bile salts probably by maintaining the cell membrane integrity [52]. It has been reported in some lactobacilli that genes encoding *SrtA* and *MucBP* proteins are overexpressed after bile exposure [53,54,55] suggesting their involvement in bile salt stress resistance. MCC0200 genome harboured both sortase A and sortase-dependent proteins (SDPs: fig|6666666.935801.peg.992, fig|6666666.935801.peg.1848), which might be involved in bile tolerance.

Besides their role as detergents, bile salts are recognized to induce oxidative stress on bacteria by generating reactive oxygen/nitrogen species [51]. Genes associated with general stress responses (*HtrA, DnaK, GroEL*) are also known to provide protection against bile stress. These protective genes were identified in MCC0200 genome.

#### 3.3.2. Adhesion Potential of MCC0200

The adhesion ability of a probiotic bacterium is a desirable characteristic, as it can extend the duration of bacterial presence in the gut, enhance the competitive exclusion of pathogens, and facilitate interactions with host surfaces. These interactions, in turn, contribute to the modulation of immune responses, delivering benefits to the host [56]. In the present investigation, a comparative study of bacterial adhesion to ECM components and cell line was performed to assess MCC0200′s ability to colonize and reside in the gut.

##### Assays for Evaluating Bacterial Adhesion

Adhesion has been linked to auto-aggregation and the hydrophobic properties of the cell surface. These assays were employed to obtain a more comprehensive understanding of the factors influencing bacterial adhesion.

1. Cell surface Hydrophobicity of MCC0200

**Figure 2:**
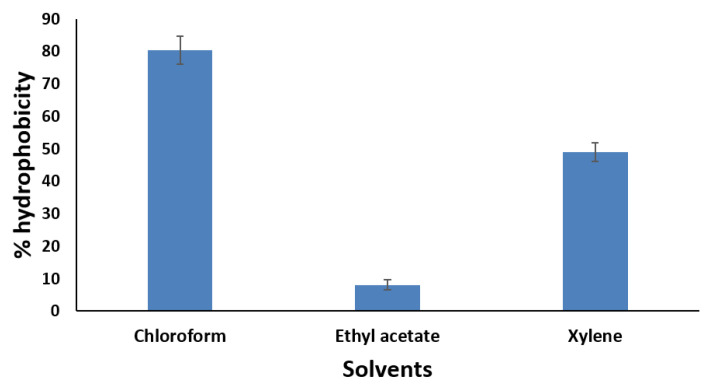
Adherence of *S. thermophilus* MCC0200 to the hydrocarbons: chloroform, ethyl acetate and xylene (the BATH method was employed to measure the hydrophobicity of the cell surface of MCC0222. This method assesses hydrophobicity as the affinity of microorganisms to hydrocarbons).

MCC0200 was evaluated for its cell surface hydrophobicity (CSH) towards different hydrocarbons, i.e., xylene, chloroform, and ethyl acetate, to assess the colonization potential of the organism to intestinal surface. As evident from Figure 2, MCC0200 exhibited affinity to all the solvents tested, ranging from 8.08 to 80.4%. Notably, it exhibited the highest hydrophobicity (80.4%) with chloroform, indicative of its strong adhesion capacity. Previous studies have suggested a strong association between bacterial cells with high hydrophobicity and their adherence to epithelial or mucous layers [57]. Hydrophobicity studies specific to *S. thermophilus* are limited, with only a study by Iyer et al., 2010 [2], reporting % hydrophobicity values in the range of 12–24.5% for different hydrocarbons.

2. Aggregation ability of MCC0200

**Figure 3:**
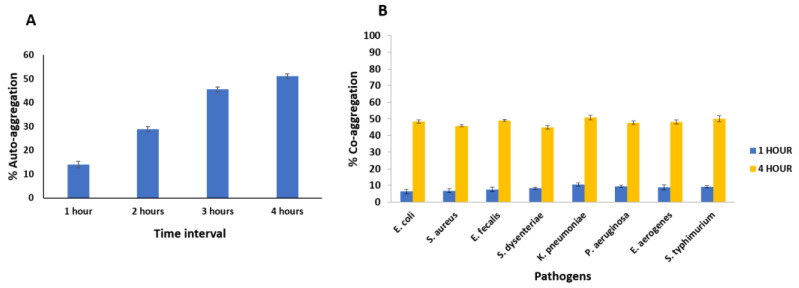
(**A**): % Auto-aggregation of MCC0200 at different time intervals; (**B**): % Co-aggregation of MCC0200 with different pathogens after 1 h and 4 h of incubation at 37 °C. Average value and ± SD from three experiments.

Auto-aggregation serves as the initial step in the adhesion process, allowing bacteria to create a barrier and hinder the adhesion of undesirable bacteria. A high auto-aggregation ability is typically defined as greater than 40%, while any strain with less than 10% is considered to have weak auto-aggregation [58]. MCC0200 exhibited the highest auto-aggregation capacity of 51.1% after 4 h (Figure 3A). This indicated the potential of the isolate to colonize the intestinal epithelium once adhesion has been established. A study by Taj et al., 2022 [59], has reported auto-aggregation percentages of 97.8 ± 0.4, 61.2 ± 1.0, and 53.6 ± 0.6 for different strains of *S. thermophilus*. Tuncer and Tuncer (2014) [49] reported 49.55 ± 6.24% auto-aggregation *of S. thermophilus* ST8.01 strain, which was found to be comparable with our findings.

Probiotics can exclude or reduce the growth of other microorganisms in the intestine through competition for nutrients or adherence sites. Regarding the potential mechanisms of pathogen exclusion, one plausible action is the co-aggregation of probiotics with pathogenic bacteria, which could prevent the attachment of pathogens to the intestinal surface and impede their colonization in humans [60]. MCC0200 demonstrated the ability to co-aggregate with all tested pathogenic strains. The maximum co-aggregation potential of MCC0200, reaching up to 50%, was observed with *K. pneumoniae* and *S. typhimurium* (Figure 3B). Interactions between carbohydrate-lectin and proteinaceous components present on the cell surface may be implicated in the co-aggregative properties of *Streptococcus* sp. [61].

Surface hydrophobicity, auto-aggregation, and co-aggregation properties collectively suggested the robust adhesion and colonization potential of MCC0200, which was further validated with intestinal cell adhesion assays.

3. Adhesion to mucin, fibrinogen and collagen

Ability to bind to the extracellular matrix (ECM) is recognized as a characteristic of many pathogenic bacteria. Conversely, probiotic bacterial strains with this binding capability may compete with pathogens for the same receptors and occupy potential binding sites in the gut. Consequently, adhesion to the mucosal surface stands as a crucial prerequisite for the colonization of probiotic organisms in the gastrointestinal tract, providing these organisms a competitive advantage in the gut [62]. MCC0200 was investigated for its ability to bind to ECM molecules (mucin, collagen and fibrinogen).

**Figure 4:**
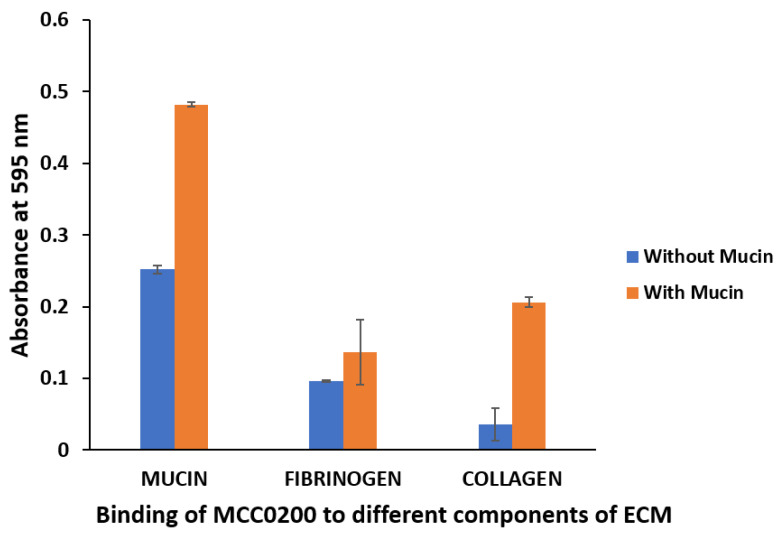
Binding of MCC0200 to different ECM substrates (mucin, fibrinogen, and collagen) immobilized in microtitre plates.

MCC0200 showed significant binding to mucin (Figure 4). It has been described that the adherence to mucus in several probiotic strains is an inducible characteristic triggered by the presence of mucin in the growth medium [63]. To assess this characteristic feature, MCC0200 was grown in the presence of mucin and tested for mucin binding. The inclusion of mucin had a significant impact on the adhesion capability of MCC0200, resulting in an increased binding to the ECM components. However, MCC0200 had an intermediate binding ability to fibrinogen and showed the lowest adherence to collagen in absence of mucin. Fernandez et al., 2018 [64], studied the mucus-related properties of *S. thermophilus* (LMD-9 and LMG18311). The two strains displayed weak binding to mucus/mucins (<0.1) relative to the highly adhesive TIL448 *Lactococcus lactis*, characterizing *S. thermophilus* as a poor mucus-adhesive bacterium. Our findings deviated from the reported study, wherein, *S. thermophilus* MCC0200 used in the present study showed strong binding to mucin (OD~0.48).

##### Adhesion of MCC0200 to HT-29 Cell Line

The adherence of micro-organisms to biological surfaces is a crucial criterion when selecting potential probiotic strains [65]. In the present study, human colonic adenocarcinoma, HT-29 cell line, which express structural and functional features similar to normal human enterocytes [66], was utilized as in vitro model.

**Figure 5:**
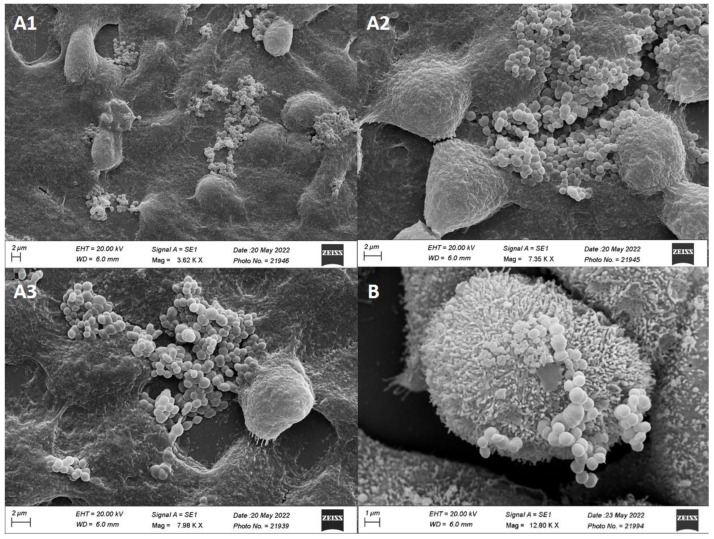
(**A1**–**A3**). Adhesion of MCC0200 on HT-29 cells (magnification level 3.62 k× to 7.98 k×) and (**B**): MCC0200 adhering to microvilli that form the brush border of HT-29 monolayer observed under the scanning electron microscope (magnification level 12.80 k×).

MCC0200 was found to be well adherent to HT-29 human colonic cells (Figure 5A1–A3). In particular, 638 ± 37 MCC0200 cells adhered per 100 HT-29 cells. The secretion of mucus by the cells may have a substantial impact on the adhesion process. The adhesive pattern of MCC0200 appeared to be localized and in clusters, aligning with its high auto-aggregative property (51%).

The evaluation of the adhesive phenotype was conducted in accordance with the observations documented by Haeri et al. in 2012 [67], categorizing bacteria as: (a) poorly adhesive: with less than 20 bacterial cells adhered per 100 animal cells, (b) moderately adhesive: with 21 to 50 bacteria adhered per 100 animal cells, and (c) strongly adhesive: with more than 51 bacteria adhered per 100 animal cells. In our study, MCC0200 was found to be strongly adherent to HT-29 cell line.

Human adenocarcinoma cells undergo spontaneous differentiation, displaying structural and functional polarization and differentiation. At late confluency these cells develop brush border microvilli structures and produce mucin [68]. Scanning electron microscopy revealed the presence of a dense and well-organized brush border microvilli structures on HT-29 cells. These microvilli were the attachment sites for MCC0200, as observed in Figure 5B.

A study by Fernandez et al., 2018 [64] revealed *S. thermophilus* to be a poorly adhesive bacterium relative to other mucus-adhesive lactic acid bacteria (*Lactobacillus reuteri* and *Lactobacillus plantarum*). Adhesion property was found to be not the most determinant trait of *S. thermophilus*. However, the strain MCC0200 of *S. thermophilus* used in our study was found to be strongly adherent to human adenocarcinoma cell lines.

Furthermore, genome mining of MCC0200 unveiled a cascade of adhesion-related genes (Table 4). Predicted fibronectin binding protein (*FnBP*) suggested MCC0200’s ability to bind to fibronectin, a cell-surface dimeric glycoprotein. Kapczynski et al., 2000 [69], reported a correlation between fibronectin binding and adherence of bacteria to intestinal cells in vitro corroborated subsequent in vivo relationship. Strain MCC0200 possessed a functional sortase A involved in anchoring of LPxTG-cell wall proteins [52], indicative of its role in interactions with intestinal epithelial cells and/or mucus components. Additionally, several moonlighting proteins such as enolase, *EF-Tu*, *EF-G*, triosephosphate isomerase, *GroEL*, *DnaK*, pyruvate kinase, inosine 5′-monophosphate dehydrogenase *(IMPDH)*, glutamine synthetase and glucose-6-phosphate isomerase *(GPI)* were detected in MCC0200 genome. These moonlighting proteins perform adhesive functions, interacting with host epithelial cells, mucus, extracellular matrix (ECM) components, and circulating host components [70]. Eleven genes encoding EPS biosynthesis namely, glycosyltransferases, *epsA*, *epsC*, and *epsD* were also mapped in MCC0200 genome, which might assist its adhesion to intestinal mucus.

#### 3.3.3. Antioxidant Activity

Studies suggest that some probiotic bacteria can counteract the detrimental effects of oxidative stress by scavenging the reactive oxygen species (ROS), thereby maintaining the redox balance in the gut [71]. The antioxidant capacity of the MCC0200 was found to be 45.06 ± 3.64% of DPPH scavenging activity and 93.03 ± 0.03% of ABTS scavenging activity. The ABTS scavenging activity of MCC0200 was highest as compared to that of ascorbic acid (of 69.49%), indicative of MCC0200′s excellent antioxidative potential. The disparity in radical scavenging activity between DPPH and ABTS may be attributed to differences in solubility and diffusivity in the substrate. Notably, our strain MCC0200 demonstrated more robust antioxidant activity compared to reported values for other strains of *S. thermophilus*. Various studies have documented diverse ABTS scavenging activities of 56.6% (EPS from *S. thermophilus* CRL1190) and 27.1% for different *S. thermophilus* strains [72,73].

##### The Redox System in MCC0200

Genome analysis revealed the redox system operative in MCC0200 contributing to its radical scavenging activity. The genes identified in the MCC0200 genome that potentially aid in tolerance to oxidative stress are summarized in Table 5. Gene encoding superoxide dismutase (*SOD*), particularly *MnSOD* was detected in MCC0200 genome. MnSODs play a role in reducing the level of O^2^, thereby contributing to anti-oxidative activity via iron chelation [74]. However, the absence of genes encoding catalase suggests that the glutathione and thioredoxin systems primarily function in detoxifying hydrogen peroxide generated by *SOD*. The presence of genes encoding the thioredoxin–thioredoxin reductase system (*Trxs*) and glutathione–glutaredoxin system (*Grxs*) in MCC0200 genome implicates another mechanism of redox homeostasis, regulating the thiol–disulfide balance. This system is crucial for resisting the toxic effects of hydrogen peroxide. Additionally, thiol-dependent peroxidases (peroxiredoxins) were detected, which are actively involved in eliminating reactive oxygen and nitrogen species. The *MSRA/B*-mediated oxidation and reduction in methionine residues represent another important antioxidant mechanism detected in MCC0200. The *Msr* system is known to prevent irreversible protein damage and contribute to cellular antioxidant resistance, thus extending the organism’s lifespan [75]. 

Further auxiliary protective systems in MCC0200 to withstand oxidative stress include genes involved in the repair of damaged proteins and DNA. MCC0200 genome harboured genes encoding *recA*, chaperonins: *DnaK* and *GroEL*, *Hrc*, *HtrA, Clp proteases*, universal stress proteins.

Overall, these findings suggested the antioxidative potential of MCC0200 and its ability to provide ROS protective factors to host.

#### 3.3.4. MCC0200 as Nutrient Factory: Biosynthetic Capabilities

Probiotics capable of producing vital vitamins could be regarded as nutritive supplements for individuals lacking adequate levels of these vital nutrients. The genome of MCC0200 comprises genes required for synthesis of vitamin B9 (folate). The genes detected in MCC0200 that are involved in the folate biosynthesis are illustrated in Table 6.

**Figure 6:**
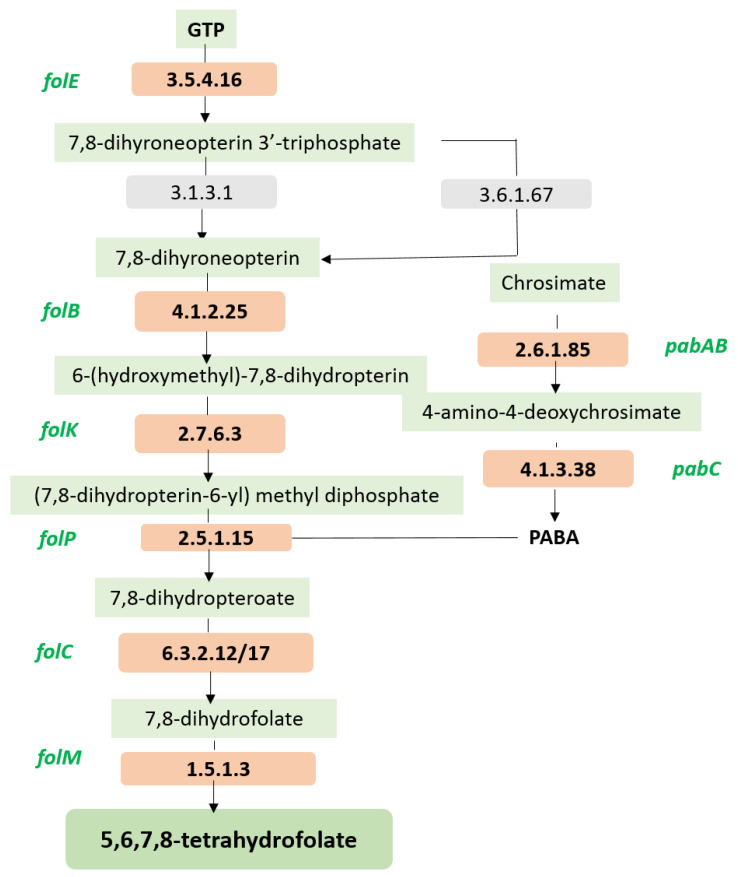
Folate biosynthesis pathway in MCC0200 mapped using KEGG database.

Folate (vitamin B9) serves as a cofactor, in various crucial cellular functions, including the synthesis of nucleic acids, amino acids, cellular growth, and cell division. Deficiency in folate is linked to several human health disorders, including osteoporosis, impaired cognitive performance, Alzheimer’s disease, neural tube defects in newborns, etc. [2]. Since humans cannot synthesize folate intrinsically, it must be obtained through dietary sources. Interestingly, the folate content in food products can increase significantly, up to 20-fold, following fermentation by folate-producing bacteria such as *Streptococcus thermophilus* [76]. Folate is synthesized from precursors GTP and p-aminobenzoate (PABA), originating from purine and phenylalanine metabolism, respectively. In MCC0200 genome, the entire pathway for folate synthesis was mapped (Figure 6) using the Kyoto Encyclopedia of Genes and Genomes (KEGG) database, shedding light on its potential application in enhancing folate levels for nutritional benefits.

#### 3.3.5. Beta Galactosidase Production

Lactose intolerance is a medical condition characterized by a deficiency of the enzyme beta-galactosidase, resulting in the inability to hydrolyse lactose into the monosaccharides glucose and galactose. Symptoms like diarrhoea, abdominal discomfort, and flatulence may arise following the consumption of milk or milk products. There are assertions that probiotic cultures with increased beta-galactosidase activity could potentially aid individuals with lactose intolerance in enhancing their lactose metabolism. MCC0200 demonstrated a colour change on CLED agar, indicating its ability to utilize lactose through the production of beta-galactosidase. The gene encoding beta-galactosidase (fig|6666666.935801.peg.1886) was identified in the MCC0200 genome. The likely mechanism for lactose utilization includes the transport of lactose through the permease system, followed by its hydrolysis by β-galactosidase to produce glucose and galactose. Additionally, β-galactosidase can catalyse the transgalactosylation of lactose into allolactose [77]. Subsequent to allolactose synthesis, β-galactosidase can polymerize the disaccharide into galactooligosaccharides. These galactooligosaccharides are regarded as prebiotics, and there is considerable contemporary research focused on identifying specific microorganisms that yield high quantities of GOS [78].

Thus, MCC0200, with its β-galactosidase activity, holds potential benefits in mitigating the effects of lactose intolerance. It could also find emerging applications in producing beverages enriched with prebiotics such as GOS, leveraging the multifunctional enzymatic nature of β-d-galactosidase.

#### 3.3.6. In Vitro Evaluation of the Anti-Hypercholesterolemic Effect of MCC0200

The supplementation of probiotics with cholesterol-lowering capacities has been suggested as a viable strategy to decrease serum cholesterol. Several proposed mechanisms for probiotic-mediated cholesterol reduction include: (a) deconjugation of bile acids via BSH, (b) integration of cholesterol into the bacterial membrane-phospholipid bilayer through adherence to the cell surface, (c) cholesterol assimilation by growing cells, (d) cholesterol co-precipitation with a deconjugated bile salt, and (e) transformation of cholesterol to coprostanol [79]. However, there is still limited understanding of the mechanistic insights into cholesterol reduction by probiotic bacteria.

In the present study, the anti-hypercholesterolemic ability of MCC0200 was assessed in SIF. MCC0200 demonstrated the assimilation of 43.01 ± 5.44% cholesterol in the SIF after 24 h of incubation. Considering the significance of cholesterol in cardiovascular disease and related illnesses, MCC0200, with its cholesterol-removing ability, may emerge as a potential candidate for applications in food and pharmaceuticals. Ziarno (2010) [80] evaluated the cholesterol-lowering activity of 12 strains of *S. thermophilus*, ranging from 2.2% to 6.3% after 6 h of incubation at 37 °C.

Bile salt hydrolase (*BSH*) activity is recognized as a significant marker linked to hypocholesterolaemic effects [79]. However, the absence of candidate gene encoding BSH in the MCC0200 genome suggests the involvement of alternative mechanisms in lowering cholesterol. Several studies propose that cholesterol is either integrated into bacteria or adheres to the bacterial cell surface. Noh et al., 1997 [81] hypothesized that cholesterol incorporated into bacterial cells alters the cell membrane or cell wall. According to this hypothesis, membrane-associated proteins are implicated in playing a crucial role in the process of cholesterol reduction. The hypocholesterolaemic impact of MCC0200 may be attributed to the presence of cholesterol reduction-related proteins, including a transcription regulator, fructose bisphosphate aldolase (two copies), catabolite control protein A (*ccpA*) gene, and *MFS* in MCC0200. Additionally, the *ccpA* gene and its associated proteins may contribute to cholesterol reduction in MCC0200 through cell membrane modulation. However, the findings of this experiment require validation through targeted gene mutation experiments. A study by Lee et al., 2010 [82], highlighted the significant role of *ccpA*, encoding catabolite control protein A, in cholesterol reduction by probiotic bacteria (*L. acidophilus* A4).

### 3.4. Safety Assessment of MCC0200 as a Probiotic

Even though probiotics are considered safe for consumption, there are certain concerns that need to be addressed before any organism or strain is selected as a probiotic. Key safety considerations for probiotics include: (a) ensuring that the strain does not cause any diseases like endocarditis or bacteraemia, (b) should not produce toxins or metabolites that could harm the gastrointestinal tract, and (c) should be free of plasmids or transposable elements that might facilitate the transfer of antibiotic resistance determinants to the gastrointestinal flora [83].

The E strip test revealed that MCC0200 was susceptible to ampicillin, vancomycin, streptomycin, clindamycin, tetracycline, and chloramphenicol, as per the established cut-off provided by EFSA FEEDAP Panel, 2018.

#### Genome Based Safety Evaluation of MCC0200

The whole genome sequence of MCC0200 was mined to check for the presence of genes for antibiotic resistance using CARD and ResFinder. There were no antibiotic resistance genes detected in the genome of MCC0200 indicating the GRAS nature of *S. thermophilus* MCC0200. For the presence of bacteriophage, the PHASTER tool identified four incomplete prophage regions in the main chromosome. The absence of plasmids was confirmed by Plasmid Finder (v2.0.1). None of the genes were associated with plasmids, and the risk of transfer was ranked at the lowest degree. No virulence factors or toxin encoding genes were identified in the genome of MCC0200 using VirulenceFinder.

The genome sequence MCC0200 was also mined for the presence of toxin genes using known toxin nucleotide sequences as a reference and MCC0200 as query. The bceT gene, which encodes the single-component enterotoxin T, and the haemolytic enterotoxin hbl, known to carry three genes (hblA, hblB and hblC) were not detected. Non-haemolytic enterotoxin (Nhe) which codes for three genes (nheA, nheB and nheC) was also not observed in MCC0200. Thus, the results of this analysis did not yield any hits which suggested the absence of any emetic toxin genes in the genome of MCC0200.

The CRISPR Finder software (https://crisprcas.i2bc.paris-saclay.fr (accessed on 26 July 2022)) was used to search for CRISPR direct repeats and spacers. Two CRISPR genes, one Cas gene and 25 spacers were detected. Collectively, these analyses indicate the safe nature of MCC0200 for human consumption.

## 4. Conclusions

The present study was undertaken to demonstrate the probiotic intricacies of *Streptococcus thermophilus* MCC0200. In particular, the traits essential for a probiotic to thrive through the gastrointestinal transit and colonize the gut were established at physiological as well as molecular level. Moreover, the strong antioxidant capacity of MCC0200 could be involved in controlling and preventing several chronic illnesses related with oxidative stress. MCC0200 could also be used as an alternative supplement to confer health benefits, such as lowering cholesterol levels and alleviating lactose intolerance. The safety evaluation of MCC0200 indicated that it did not harbour any antibiotic resistance genes and that it is sensitive to multiple antibiotics. In vitro studies corroborated with in silico studies proved MCC0200 as a potential probiotic, paving the way for further investigations and potential applications beyond its traditional role in the dairy industry.

## Figures and Tables

**Table 1:** Genome features of *S. thermophilus* MCC0200.

Genome Attributes	Values
Genome size (bp)	1,855,815
GC content %	39.1
Number of contigs	6
Protein coding genes (CDS)	2239
Subsystems	219
RNA encoding genes	83

**Table 2:** DNA–DNA hybridization values and ANI values between query (MCC0200) and other *S. thermophilus* reference genomes.

Reference Strain	% ANI	% DDH
*Streptococcus thermophilus* LMD-9	99.93	99.7
*Streptococcus thermophilus* TH1477	98.96	91.7
*Streptococcus thermophilus* MTH17CL396	99.01	92.2
*Streptococcus thermophilus* TH1436	99.25	94
*Streptococcus thermophilus* TH1435	99.31	93.9

**Table 3:** Putative genes encoding proteins involved in acid and bile salt tolerance detected in MCC0200 genome.

Genes Detected in MCC0200	FigFam No.	Predicted Function
ATP synthase subunit a	fig|6666666.935801.peg.921	
ATP synthase subunit b	fig|6666666.935801.peg.922	Acid tolerance by maintaining pH homeostasis
ATP synthase subunit c	fig|6666666.935801.peg.920	
ATP synthase alpha chain	fig|6666666.935801.peg.924	
ATP synthase Beta chain	fig|6666666.935801.peg.926	
ATP synthase Gamma chain	fig|6666666.935801.peg.925	
ATP synthase Epsilon chain	fig|6666666.935801.peg.927	
ATP synthase delta chain	fig|6666666.935801.peg.923	
Na^+^/H^+^ antiporter	fig|6666666.935801.peg.2147	
Urease system Urease cluster protein	fig|6666666.935801.peg.706	Acid tolerance by Alkali production
Alpha	fig|6666666.935801.peg.707	
Beta	fig|6666666.935801.peg.711	
Gamma	fig|6666666.935801.peg.710	
Accessory proteins:	fig|6666666.935801.peg.709	
Urease accessory protein UreD	fig|6666666.935801.peg.715	
Urease accessory protein UreE	fig|6666666.935801.peg.712	
Urease accessory protein UreF	fig|6666666.935801.peg.713	
Urease accessory protein UreG	fig|6666666.935801.peg.714	
Ffh	fig|6666666.935801.peg.1370	Proteins involved in protection and repair of molecules under acid stress
DnaK	fig|6666666.935801.peg.487	
DnaJ	fig|6666666.935801.peg.488	
GrpE	fig|6666666.935801.peg.485	
HrcA	fig|6666666.935801.peg.484	
GroEL	fig|6666666.935801.peg.603	
GroES	fig|6666666.935801.peg.601	
Clp proteases	fig|6666666.935801.peg.801	
EF-Tu	fig|6666666.935801.peg.929	
recA	fig|6666666.935801.peg.410	
recN	fig|6666666.935801.peg.1668	
Exonuclease V	fig|6666666.935801.peg.1681	
UvrABCD	fig|6666666.935801.peg.31	
	fig|6666666.935801.peg.1985	
	fig|6666666.935801.peg.1783	
	fig|6666666.935801.peg.1458	
DNA polymerase	fig|6666666.935801.peg.46	
DNA ligase	fig|6666666.935801.peg.2048	
Sortase A	fig|6666666.935801.peg.1755	Proteins involved in bile salt tolerance
Sortase-dependent proteins	fig|6666666.935801.peg.992	
	fig|6666666.935801.peg.1848	
HtrA	fig|6666666.935801.peg.349	
DnaJ	fig|6666666.935801.peg.488	
GroEL	fig|6666666.935801.peg.603	

**Table 4:** Presence of genes involved in colonization of the intestinal mucosa detected in MCC0200 genome.

Genes Detected in MCC0200	Predicted Function	FigFam No.
Fibronectin/fibrinogen-binding protein	Binds to fibronectin	fig|6666666.935801.peg.1423
Sortase A, LPXTG specific	Cell surface localization and peptidoglycan interaction	fig|6666666.935801.peg.1755
**Moonlighting proteins**		
Enolase	Binding to plasmin(ogen), fibronectin, laminin, albumin, collagen, salivary mucin, intestinal epithelial cells,	fig|6666666.935801.peg.1108
*EF-Tu*	Binding to plasmin(ogen), plasma Factor H and Factor H-related protein 1 (FHR-1), intestinal epithelial cells and HT-MTX-derived mucus, salivary mucin, fibronectin	fig|6666666.935801.peg.929
*EF-G*	Binding to salivary mucin	fig|6666666.935801.peg.75
Triosephosphate isomerase	Binding to plasmin(ogen), intestinal epithelial cells,	fig|6666666.935801.peg.930
*GroEL*	Binding to intestinal HT-29 cells and mucus	fig|6666666.935801.peg.603
*DnaK*	Binding to plasmin(ogen)	fig|6666666.935801.peg.486
Pyruvate kinase	Binding to salivary mucin	fig|6666666.935801.peg.1651
Inosine 5′-monophosphate dehydrogenase *(IMPDH)*	Binding to plasmin(ogen)	fig|6666666.935801.peg.342
Glutamine synthetase	Binding to plasmin(ogen), laminin, collagen I, fibronectin	fig|6666666.935801.peg.61
Glucose-6-phosphate isomerase *(GPI)*	Binding to collagen	fig|6666666.935801.peg.585

**Table 5:** Putative genes encoding oxidative stress proteins detected in MCC0200 genome.

Gene Detected in MCC0200 Genome	FigFam No.	Predicted Function
Thiol peroxidase, Tpx-type (EC 1.11.1.15)	fig|6666666.935801.peg.1462	H_2_O_2_-degrading enzymes
NADH peroxidase	fig|6666666.935801.peg.1758	
Superoxide dismutase [Mn] (EC 1.15.1.1)	fig|6666666.935801.peg.1192	Hydroperoxide radical detoxification
Thioredoxin reductase (EC 1.8.1.9)	fig|6666666.935801.peg.1905	Redox homeostasis
Thioredoxin	fig|6666666.935801.peg.88	
Peptide-methionine (S)-S-oxide reductase *MsrA/MrsB*	fig|6666666.935801.peg.1824	Resistance to oxidative stress
	fig|6666666.935801.peg.2133	
*recA*	fig|6666666.935801.peg.410	Induces DNA repair mechanism
*GroES/EL,* clp proteases, *CtsR*, *HrcA*	fig|6666666.935801.peg.602 fig|6666666.935801.peg.603 fig|6666666.935801.peg.801 fig|6666666.935801.peg.425	Targeting and degradation of misfolded proteins.
	fig|6666666.935801.peg.484	
*HtrA*	fig|6666666.935801.peg.349	Proteolysis of abnormal proteins
*GrpE*	fig|6666666.935801.peg.485	Proper protein folding

**Table 6:** Vitamin biosynthetic proteins/genes detected in MCC0200.

Folate Biosynthesis Protein/Gene/System Detected in the MCC0200	FigFam No.
*FolE,* GTP cyclohydrolase I (EC 3.5.4.16) type 1	fig|6666666.935801.peg.2035
*FolB,* dihydroneopterin aldolase	fig|6666666.935801.peg.2032
*FolK*,2-amino-4-hydroxy-6-hydroxymethyldihydropteridine pyrophosphokinase (EC 2.7.6.3)	fig|6666666.935801.peg.2031
*FolP,* Dihydropteroate synthase (EC 2.5.1.15)	fig|6666666.935801.peg.2034
*FolC1,* Dihydrofolate synthase (EC 6.3.2.12)	fig|6666666.935801.peg.846
*FolC2,* Dihydrofolate synthase (EC 6.3.2.12)	fig|6666666.935801.peg.2038
*FolM*, FolA, Dihydrofolate reductase (EC 1.5.1.3)	fig|6666666.935801.peg.1044
*PabC,* Aminodeoxychorismate lyase	fig|6666666.935801.peg.1232
*PabAB*, Para-aminobenzoate synthase, aminase component (EC 2.6.1.85)	fig|6666666.935801.peg.1232

## Data Availability

Genome sequence is available in NCBI GenBank under the accession number JAVCAM010000000.

## Supplementary Materials

The following supporting information can be downloaded at: https://www.mdpi.com/article/10.3390/microorganisms12020347/s1, Figure S1. MLST tree constructed based on various housekeeping genes by autoMLST. The highlighted sequence is the query sequence of MCC0200. Table S1. Ability of MCC0200 to survive in Simulated Gastric fluid at different time intervals; Table S2 Ability of MCC0200 to survive in Simulated Intestinal fluid at different time intervals.
