# Supplementary material for: Unveiling the Probiotic Potential of Streptococcus thermophilus MCC0200: Insights from In Vitro Studies Corroborated with Genome Analysis
Source: Microorganisms. 2024 Feb 7;12(2):347. doi: 10.3390/microorganisms12020347 (PMC10891967; doi:10.3390/microorganisms12020347)
Supplement: Supplementary file 1 [file microorganisms-12-00347-s001.zip › microorganisms-2787720-supplementary.pdf]

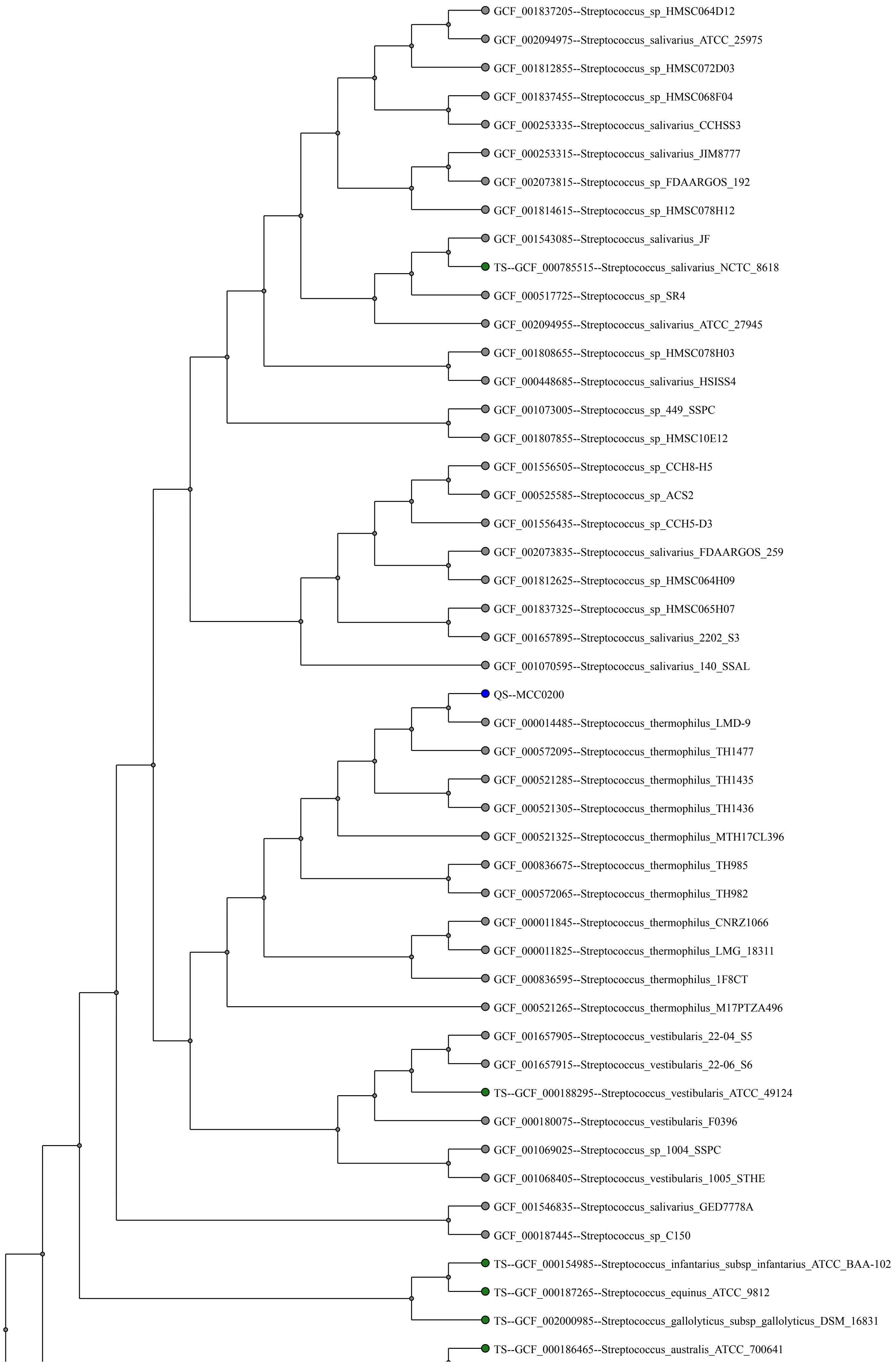
GCF_001837205--Streptococcus_sp_HMSC064D12
GCF_002094975--Streptococcus_salivarius_ATCC_25975
GCF_001812855--Streptococcus_sp_HMSC072D03
GCF_001837455--Streptococcus_sp_HMSC068F04
GCF_000253335--Streptococcus_salivarius_CCHSS3
GCF_000253315--Streptococcus_salivarius_JIM8777
GCF_002073815--Streptococcus_sp_FDAARGOS_192
GCF_001814615--Streptococcus_sp_HMSC078H12
GCF_001543085--Streptococcus_salivarius_JF
TS--GCF_000785515--Streptococcus_salivarius_NCTC_8618
GCF_000517725--Streptococcus_sp_SR4
GCF_002094955--Streptococcus_salivarius_ATCC_27945
GCF_001808655--Streptococcus_sp_HMSC078H03
GCF_000448685--Streptococcus_salivarius_HSISS4
GCF_001073005--Streptococcus_sp_449_SSPC
GCF_001807855--Streptococcus_sp_HMSC10E12
GCF_001556505--Streptococcus_sp_CCH8-H5
GCF_000525585--Streptococcus_sp_ACS2
GCF_001556435--Streptococcus_sp_CCH5-D3
GCF_002073835--Streptococcus_salivarius_FDAARGOS_259
GCF_001812625--Streptococcus_sp_HMSC064H09
GCF_001837325--Streptococcus_sp_HMSC065H07
GCF_001657895--Streptococcus_salivarius_2202_S3
GCF_001070595--Streptococcus_salivarius_140_SSAL
QS--MCC0200
GCF_000014485--Streptococcus_thermophilus_LMD-9
GCF_000572095--Streptococcus_thermophilus_TH1477
GCF_000521285--Streptococcus_thermophilus_TH1435
GCF_000521305--Streptococcus_thermophilus_TH1436
GCF_000521325--Streptococcus_thermophilus_MTH17CL396
GCF_000836675--Streptococcus_thermophilus_TH985
GCF_000572065--Streptococcus_thermophilus_TH982
GCF_000011845--Streptococcus_thermophilus_CNRZ1066
GCF_000011825--Streptococcus_thermophilus_LMG_18311
GCF_000836595--Streptococcus_thermophilus_1F8CT
GCF_000521265--Streptococcus_thermophilus_M17PTZA496
GCF_001657905--Streptococcus_vestibularis_22-04_S5
GCF_001657915--Streptococcus_vestibularis_22-06_S6
TS--GCF_000188295--Streptococcus_vestibularis_ATCC_49124
GCF_000180075--Streptococcus_vestibularis_F0396
GCF_001069025--Streptococcus_sp_1004_SSPC
GCF_001068405--Streptococcus_vestibularis_1005_STHE
GCF_001546835--Streptococcus_salivarius_GED7778A
GCF_000187445--Streptococcus_sp_C150
TS--GCF_000154985--Streptococcus_infantarius_subsp_infantarius_ATCC_BAA-102
TS--GCF_000187265--Streptococcus_equinus_ATCC_9812
TS--GCF_002000985--Streptococcus_gallolyticus_subsp_gallolyticus_DSM_16831
TS--GCF_000186465--Streptococcus_australis_ATCC_700641

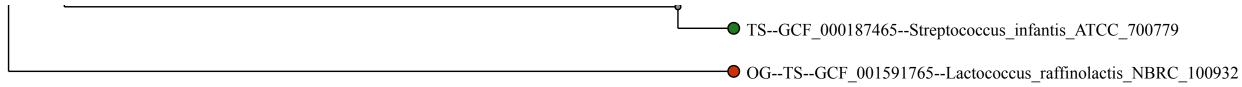

Figure S1. MLST tree constructed based on various housekeeping genes by autoMLST. The highlighted sequence is the query sequence of MCC0200.

Table S1. Ability of MCC0200 to survive in Simulated Gastric fluid at different time intervals

**Time interval (min)**	**Total Viable Count of MCC0200 (CFU/ml)**
**0**	$3.75 \pm 10 \times 10^{7}$
**30**	$2 \pm 0 \times 10^{4}$
**60**	$1.5 \pm 0 \times 10^{2}$

Table S2. Ability of MCC0200 to survive in Simulated Intestinal fluid at different time intervals

**Time interval (min)**	**Total Viable Count of MCC0200 (CFU/ml)**
**0**	$1.22 \pm 1.76 \times 10^{8}$
**30**	$6.5 \pm 0.84 \times 10^{7}$
**60**	$5.85 \pm 0.07 \times 10^{7}$
**120**	$2.3 \pm 0 \times 10^{7}$
**240**	$1.45 \pm 0.07 \times 10^{7}$
**360**	$1.1 \pm 0.28 \times 10^{7}$
